# The comparison of clinical, laboratory, and radiological findings in immunocompromised and immunocompetent patients with COVID‐19: A case‐control study

**DOI:** 10.1002/iid3.806

**Published:** 2023-04-12

**Authors:** Ladan Abasian, Fatemeh Jafari, SeyedAhmad SeyedAlinaghi, Niloofar Ayoobi Yazdi, Morteza Daraei, Nasrin Ahmadinejad, Fereshteh Ghiasvand, Hosein Khalili, Arash Seifi, Mohsen Maydani, Farzane Behnezhad, Zahra Ahmadinejad

**Affiliations:** ^1^ Department of Infectious Diseases, Imam Khomeini Hospital Complex Tehran University of Medical Sciences Tehran Iran; ^2^ Iranian Research Center for HIV/AIDS (IRCHA), Imam Khomeini Hospital Complex Tehran University of Medical Sciences (TUMS) Tehran Iran; ^3^ Department of Radiology, Imam Khomeini Hospital Complex Tehran University of Medical Sciences Tehran Iran; ^4^ Internal Medicine‐Department of Internal Medicine, Imam Khomeini Hospital Complex Tehran University of Medical Sciences Tehran Iran; ^5^ Department of Virology, School of Public Health Tehran University of Medical Sciences Tehran Iran; ^6^ Liver Transplantation Research Center, Imam Khomeini Hospital Complex Tehran University of Medical Sciences Tehran Iran

**Keywords:** clinical presentation, COVID‐19, immunosuppression, laboratory/radiological findings, SARS‐CoV‐2

## Abstract

**Background:**

Severe acute respiratory syndrome coronavirus 2 (SARS‐CoV‐2) causes coronavirus disease 2019 (COVID‐19) with significant morbidity and mortality. We reported and compared the clinical and para‐clinical findings of immunocompromised and immunocompetent COVID‐19 patients in a case‐control study at the Imam Khomeini hospital in Tehran, Iran.

**Methods:**

In this study, 107 immunocompromised COVID‐19 patients were recruited as the case group, and 107 immunocompetent COVID‐19 patients as the control group. The participants were matched based on age and sex. The patients' information was retrieved from the hospital records in an information sheet. Associations between clinical and para‐clinical findings with the immune status were assessed using bivariate and multivariate analyses.

**Results:**

The initial pulse rate and recovery time were significantly higher in immunocompromised patients (*p* < .05). Myalgia, nausea/vomiting, loss of appetite, headache, and dizziness were more frequently reported by the control group (*p* < .05). Regarding the prescribed medications' duration, Sofosbovir was used longer in the case group, while Ribavirin was used longer in the control groups (*p* < .05). The most common complication in the case group was acute respiratory distress syndrome, although no major complications were observed in the control group. According to the multivariate analysis, recovery time and Lopinavir/Ritonavir (Kaletra) prescription were significantly higher in the immunocompromised compared to the immunocompetent group.

**Conclusion:**

Recovery time was significantly longer in the immunocompromised compared to the immunocompetent group, which emphasizes the necessity of prolonged care in these high‐risk patients. Also, it is recommended to investigate the effect of novel therapeutic interventions to reduce the recovery time in addition to improving the prognosis of immunodeficient patients with COVID‐19.

## Graphical abstract

Severe acute respiratory syndrome coronavirus 2 (SARS‐CoV‐2) causes coronavirus disease 2019 (COVID‐19) with significant morbidity and mortality. We reported and compared the clinical and para‐clinical findings of immunocompromised and immunocompetent COVID‐19 patients in a case‐control study at the Imam Khomeini hospital in Tehran, Iran. A total of 107 immunocompromised COVID patients as the case group and 107 immunocompetent COVID patients as the control group was recruited in the study. The participants were matched based on age and sex. The patients' information was retrieved from the hospital records in an information sheet. Associations between clinical and para‐clinical findings with the immune status were assessed using bivariate and multivariate analyses. The clinical trial registration number is not applicable. The initial pulse rate and recovery time were significantly higher in immunocompromised patients (*p* < .05). Myalgia, nausea/vomiting, loss of appetite, headache, and vertigo were more frequently reported by the control group (*p* < .05). In terms of the duration of prescribed medications, Sofosbovir time was longer in the case group; while Ribavirin time was longer in the control groups (*p* < .05). The most common complication in the case group was acute respiratory distress syndrome although no major complications were observed in the control group. In the multivariate analysis, recovery time and Kaletra prescription were significantly higher in immunocompromised compared to the immunocompetent group. Recovery time was significantly higher in the immunocompromised compared to the immunocompetent group. This informs the current practice of dominant COVID‐19 clinical course in immunocompromised patients and communicates the related implications.

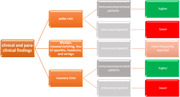

## 1 INTRODUCTION

The world is facing a new coronavirus pandemic, named severe acute respiratory syndrome coronavirus (SARS‐CoV‐2) which causes coronavirus disease 2019 (COVID‐19). It emerged in Wuhan, China, in December 2019 and soon became a pandemic.^1^ By May 29, 2020, over 5,962,944 million people had been infected, and more than 363,905 (6.10%) fatalities were reported globally.^2^ At the same time, in Iran, over 97,424 people had been infected, with a 6.83% death rate.^3^

Concerns about COVID‐19 outcomes in immunocompromised patients have been raised early in the pandemic. According to the Centers for Disease Control and Prevention guidelines, these patients were regarded as a high‐risk population for COVID‐19. There are conflicting data about COVID‐19 complications in immunocompromised hosts. Some reports demonstrated a higher risk of COVID‐19 complications and poorer prognosis in immunocompromised hosts.^3^, ^4^, ^5^, ^6^, ^7^ However, experiences with the severe acute respiratory syndrome (SARS), Middle East Respiratory Syndrome coronaviruses, and the SARS‐CoV‐2 causing COVID‐19 showed that contrary to other viral infectious diseases such as influenza, the immunosuppressed hosts are not susceptible to severe pulmonary complications of coronavirus infections, which may explain the critical role of the immune system activity in coronavirus‐induced pulmonary injury.^9^

Almost 80% of SARS‐CoV‐2 infected patients are asymptomatic or presenting mild disease.^9^, ^10^ The most common presenting symptoms in immunocompetent hosts include cough, fever, and dyspnea. Older males with underlying comorbidities, especially diabetes, hypertension, obesity, and cardiopulmonary diseases, are at higher risk of severe complications. Lymphopenia and elevated inflammatory markers are valuable laboratory findings. The most prevalent patterns in chest computed tomography are the diffuse, usually bilateral, patchy ground‐glass opacities, consolidation, and/or interstitial opacities.^11^, ^12^, ^13^, ^14^, ^15^

Initial COVID‐19 presentation in immunocompromised hosts is almost the same as in immunocompetent individuals, though it is usually less severe and is associated with a more favorable prognosis. In transplant recipients with COVID‐19, cough is more common, but nearly half are afebrile and respiratory failure is less common. Kidney transplant hosts are more prone to renal dysfunction, which might be due to the direct SARS‐CoV‐2 effect on the kidney, an immune reaction to viral antigens, or graft rejection following immunosuppressive dose reduction.^3^, ^7^, ^13^, ^16^ Despite the several reports regarding the clinical presentations of COVID‐19 in immunocompromised patients, the reports are controversial, and the differences in the clinical course with immunocompetent individuals have not been characterized. Therefore, the present study is aimed to evaluate and compare the clinical, laboratory, and radiological findings of immunocompromised and immunocompetent COVID‐19 patients.

## 2 MATERIALS AND METHODS

### 2.1 Study design and participants

In the present case‐control study, patients were grouped based on their immune system function into the case group, including immunodeficient patients with COVID‐19, and the control group, including immunocompetent patients with COVID‐19. The hospital records of 214 patients (107 immunodeficient and 107 immunocompetent patients matched for age and sex) with COVID‐19 infection were reviewed, and the related information was retrieved and recorded in an information sheet separately for each group. All inpatients with either definitive (based on positive PCR of respiratory secretions) or probable (based on clinical symptoms including sore throat, cough, myalgia, weakness, lethargy, sweating, shortness of breath, diarrhea, fever, and radiological findings including lung involvement in a chest CT scan or chest X‐ray) cases of COVID‐19 were included. The study occurred at Imam Khomeini Hospital in Tehran, Iran, in July 2020. The immunodeficient patients meeting the following criteria were considered as the case group:

- Receiving more than 10 mg of corticosteroid for more than 3 weeks.
- Long‐term low‐dose corticosteroids with a cumulative dose of 750 mg or more.
- Immunosuppressive or cytotoxic drugs other than corticosteroids, including azathioprine, methotrexate, mycophenolate mofetil (CellCept), tacrolimus (Prograf), anti‐TNF alpha, and other biologic agents over the past 6 months.
- Solid organ or bone marrow transplant.
- Hematologic malignancies with or without chemotherapy during the last 6 months.
- Solid organ malignancies with chemotherapy during the last 6 months. Human immunodeficiency virus) infection/acquired immunodeficiency syndrome.
- Primary immunodeficiencies, such as common variable immune deficiency and chronic granulomatous disease.

### 2.2 Statistical analysis

The data were analyzed using SPSS software version 22. Quantitative variables were reported as mean ± standard deviation, and qualitative variables were reported as frequency (percentage). Bivariate analysis was employed to compare the clinical and para‐clinical findings between the two groups. Logistic regression analyses, adjusting for the significant variables in bivariate analysis (*p* < .10), assessed the associations between the study parameters with the status of the immune system in two groups. The results were reported as odds ratios and 95% confidence intervals (CIs).

## 3 RESULTS

In this study, 107 immunocompromised COVID‐19 patients were recruited as the case group, and 107 immunocompetent COVID‐19 patients as the control group. The participants were matched based on age and sex. The mean age of participants was 54.4 ± 14.3 years in the immunodeficient patients with COVID‐19, and 56.6 ± 14.7 years in the immunocompetent patients with COVID‐19. Besides, in both case and control groups, 50.5% of the participants were men.

As shown in Table 1, the comparison of the vital signs and duration of the clinical course showed that the initial pulse rate and recovery time were significantly higher in immunocompromised patients (*p* < .05).

**Table 1:** The comparison of vital signs and duration of clinical course in the study groups, Imam Khomeini Hospital, Tehran, 2020.

Characteristic	Groups		*p* Value^a^
	Case mean (SD)	Control mean (SD)	
Respiratory rate	22.7 (10.9)	21.2 (5.3)	.22
Temperature	37.7 (0.82)	37.5 (0.88)	.11
Pulse rate	98.3 (16.6)	92.4 (15.6)	.008^a^
Oxygen saturation (SPO_2_)	88.8 (8.9)	90.4 (5.2)	.13
ICU length of stay (day)	10.0 (10.5)	8.3 (5.8)	.51
Recovery time	16.6 (12.5)	10.5 (8.8)	<.001^a^
The interval between the onset of the first symptom and relative recovery			
Admission time (day)	8.2 (9.6)	6.5 (6.5)	.20
Duration of symptomatic period before admission/day	7.5 (5.4)	7.8 (3.8)	.62

^a^ Independent‐samples *T*‐test.

In terms of clinical manifestations (Table 2), myalgia, nausea and vomiting, loss of appetite, headache, and dizziness were more frequently reported by the control group (*p* < .05).

**Table 2:** The comparison of clinical manifestations between the case and control groups, Imam Khomeini Hospital, Tehran, 2020.

Variables	Groups		OR (95% CI)	*p* Value
	Case *N* (%)	Control *N* (%)		
Fever	73 (68.2)	65 (60.7)	1.4 (0.79−2.43)	.25
Myalgia	52 (48.6)	71 (66.4)	0.48 (0.28−0.83)	.009*
Generalized weakness	51 (47.7)	52 (48.6)	0.96 (0.56−1.65)	.89
Cough	67 (62.6)	77 (72.0)	0.65 (0.37−1.16)	.15
Nausea & vomiting	21 (19.6)	34 (31.8)	0.52 (0.28−0.98)	.04*
Diarrhea	11 (10.3)	14 (13.1)	0.76 (0.33−1.76)	.52
Abdominal pain	6 (5.6)	15 (14.0)	0.36 (0.14−0.98)	.05
Appetite loss	37 (34.6)	52 (48.6)	0.56 (0.32−0.97)	.04*
Headache	24 (22.4)	41 (38.3)	0.46 (0.26−0.85)	.01*
Dizzines	4 (3.7)	16 (15.0)	0.22 (0.07−0.68)	.009*
Tasteless (ageusia)	6 (5.6)	9 (8.4)	0.65 (0.22−1.9)	.43
Anosmia	6 (5.6)	15 (14.0)	0.36 (0.14−0.98)	.05

Abbreviations: CI, confidence intervals; OR, odds ratios.

Table 3 describes the laboratory findings of the study groups, which shows that the first hemoglobin level was significantly higher in the control group (*p* < .05).

**Table 3:** The comparison of laboratory findings between the case and control groups, Imam Khomeini Hospital, Tehran, 2020.

Characteristic	Groups		*p* Value^a^
	Case mean (SD)	Control mean (SD)	
White blood cell (/µL)	9.3 (11.5)	69.1 (602.5)	.30
Lymphocytes (/µL)	18.3 (12.7)	19.5 (8.4)	.49
Hemoglobin (g/dL)	11.9 (2.8)	13.2 (2.1)	.001^a^
Platelet count (mL)	207.9 (106.5)	219.9 (95.8)	.40
Creatine phosphokinase (U/L)	164.8 (281.5)	233.3 (499.1)	.41
Erythrocyte sedimentation rate (mm/h)	76.7 (36.1)	70.2 (29.1)	.19
C‐reactive protein (mg/L)	110.1 (80.1)	99.2 (82.4)	.35
Creatinine (mg/dL)	1.2 (1.1)	1.2 (0.4)	.27
Aspartate aminotransferase (IU/L)	49.9 (53.5)	46.4 (41.3)	.65
Alanine transaminase (IU/L)	46.3 (41.9)	43.9 (42.4)	.75
Lactate dehydrogenase (U/L)	652.5 (363.9)	649.4 (254.9)	.96
Troponin (ng/mL)	21.9 (56.4)	52.7 (173.6)	.25
B‐type natriuretic peptide (pg/mL)	5120.7 (9748.4)	1958.9 (6050.1)	.12

^a^ Independent‐samples *T*‐test.

In terms of the duration of prescribed medication (Table 4), Sofosbuvir was administered longer in the case group, while Ribavirin was administered longer in the control groups (*p* < .05).

**Table 4:** The comparison of the medication duration between the case and control groups, Imam Khomeini Hospital, Tehran, 2020.

Characteristic	Groups		*p* Value^a^
	Case mean (SD)	Control mean (SD)	
Hydroxychloroquine time (day)	7.4 (2.6)	7.6 (2.6)	.54
Lopinavir/ritonavir (Kaletra time) (day)	6.5 (2.4)	7.5 (2.9)	.26
Atazanavir time (day)	6.6 (2.1)	6.7 (2.4)	.76
Steroid time (day)	4.1 (4.5)	6.3 (6.5)	.25
Sofosbuvir time (day)	13.1 (1.8)	9.1 (4.9)	.032^a^
Oseltamivir time (day)	4.9 (0.6)	4.9 (0.6)	.84
Ribavarin time (day)	2 (0)	5 (0)	<.001^a^
Interferon time (day)	3 (0)	3.7 (1.5)	.69
Vitamin C time (day)	2.8 (1.1)	3.7 (3.03)	.33
Intravenous immune globulin time (day)	2.6 (0.8)	2.5 (1.1)	.33

^a^ Independent‐samples *T*‐test.

Table 4 illustrates the medication type in the case and control groups. It appeared that immunocompromised patients were more likely to receive Lopinavir/Ritonavir (Kaletra) compared to the immunocompetent patients in the control group, whereas the control group received Atazanavir more commonly (*p* < .05).

Diffuse (12.2%) and anterior (77.0%) lung involvement were more prevalent among immunocompromised patients. On the other hand, in immunocompetent patients, anterior and posterior (83.9%) lung involvement was more common (Tables 5 and 6). The prevalence of complications in the case and control groups is presented in Table 7, while the comparison of medication type between the case and control groups is presented in Table 8. As shown, no complications were reported in the control group. The most common complication in the case group was acute respiratory distress syndrome (ARDS) (45%), followed by myocardial and neurological complications (20% and 25%, respectively).

**Table 5:** The prevalence of different anatomical lung involvement in radiologic examination between the case and control groups, Imam Khomeini Hospital, Tehran, 2020.

Variables	Groups		OR (95% CI)	*p* Value
	Case *N* (%)	Control *N* (%)		
Location of lung involvement				
Posterior	0 (0)	11 (12.6)	Ref	‐
Anterior	57 (77.0)	3 (3.4)	93.8 (26.3−335.18)	<0.01*
Anterior & posterior	8 (10.8)	73 (83.9)	0.023 (0.01−0.06)	<0.01*
Diffuse	9 (12.2)	0 (0)	25.4 (1.5−443.9)	0.004*

Abbreviations: CI, confidence intervals; OR, odds ratios.

**Table 6:** The pattern of lung involvement in radiologic examination between the case and control groups, Imam Khomeini Hospital, Tehran, 2020.

Characteristic	Groups		*p* Value^a^
	Case mean (SD)	Control mean (SD)	
Ground‐glass opacification/opacity (%)	68 (32.7)	53.9 (32.9)	.43
Consolidation (%)	50 (14.2)	58.3 (28.5)	.57
Ground‐glass opacity plus alveolar (mix)	68 (16.4)	67.9 (21.7)	.99

^a^ Independent‐samples *T*‐test.

**Table 7:** The prevalence of complications in the case and control groups, Imam Khomeini Hospital, Tehran, 2020.

Variables	Groups		OR (95% CI)	*p* Value
	Case *N* (%)	Control *N* (%)		
Complication				
Complications during hospitalization				
ARDS	9 (45.0)	0 (0)	20.7 (1.19−360.97)	.01^a^
Neurologic	4 (20.0)	0 (0)	9.35 (0.50−175.80)	.18
Myocarditis	5 (25.0)	0 (0)	11.54 (0.63−211.28)	.10
Sepsis	2 (10.0)	0 (0)	5.095 (0.24−107.39)	.56

Abbreviations: ARDS, acute respiratory distress syndrome; CI, confidence intervals; OR, odds ratios.

^a^ Fisher exact test.

**Table 8:** The comparison of medication type between the case and control groups, Imam Khomeini Hospital, Tehran, 2020.

Variables	Groups		OR (95% CI)	*p* Value
	Case *N* (%)	Control *N* (%)		
Hydroxychloroquine	106 (99.1)	106 (99.1)	1.0 (0.06−16.19)	>.9
Lopinavir/ritonavir(Kaletra)	49 (45.8)	11 (10.3)	7.37 (3.55−15.31)	<.001*
Atazanavir	56 (52.3)	91 (85.0)	0.19 (0.1−0.37)	<.001*
Steroid	21 (19.6)	18 (16.8)	1.21 (0.6−2.42)	.60
Sofosbuvir	9 (8.4)	11 (10.3)	0.8 (0.32−2.02)	.64
Oseltamivir	47 (43.9)	39 (36.4)	1.37 (0.79−2.36)	.27
Remdesivir	0 (0)	2 (1.9)	0.196 (0.01−4.14)	.56
Ribavirin	2 (2.0)	1 (1.0)	2.1 (0.19−23.6)	.55
Interferon	2 (2.9)	4 (4.5)	0.63 (0.113−3.57)	.61
Altebrel	0 (0)	4 (3.8)	0.11 (0.01−2.01)	.18
Fluconazole	2 (1.9)	0 (0)	5.10 (0.24−107.39)	.56
Intravenous immune globulin	7 (6.5)	9 (8.4)	0.76 (0.27−2.13)	.60

Abbreviations: CI, confidence intervals; OR, odds ratios.

In the multivariate analysis (Table 9), adjusting for other significant factors in bivariate analysis (*p* < .1), recovery time and Lopinavir/Ritonavir (Kaletra) prescription were significantly higher in the immunocompromised compared to the immunocompetent group.

**Table 9:** The final significant associated factor after adjustment; Imam Khomeini Hospital, Tehran, 2020.

Associated factors	AOR	95% CI	*p* Value
Recovery time	0.94	0.91−0.98	.001
Kaletra	4.32	1.47−12.69	.008

Abbreviations: AOR, adjusted odds ratio; CI, confidence intervals.

## 4 DISCUSSION

Life‐long immunosuppression increases the vulnerability to viral infections in immunocompromised patients.^18^ Although immunocompromised patients may be more vulnerable to SARS‐CoV‐2 infection, long‐term immunosuppressive treatment may protect them against severe COVID‐19 complications.^18^, ^19^

In our study, some clinical manifestations were more prevalent in the control group, including myalgia, nausea and vomiting, loss of appetite, headache, and dizziness. Fever (98.6%), myalgia or fatigue (69.6%), dry cough, and diarrhea were the most common presentations in COVID‐19 patients.^20^, ^21^ Although gastrointestinal symptoms are common in immunocompetent COVID‐19 patients, they are rarely reported in immunocompromised COVID‐19 patients (3%−5%).^22^, ^23^, ^24^

A higher level of initial hemoglobin was observed in the control group. Bhowmik et al. also reported a lower hemoglobin level in severe cases of COVID‐19 patients (severe vs. non‐severe, 37.73% vs. 68.27%, MD = −0.73, 95% CI = −1.61 to 0.14, *p* = .10).^26^ Laboratory parameters of COVID‐19 patients included elevated LDH (lactate dehydrogenase), prothrombin time, d‐dimer, creatine kinase, and C‐reactive protein levels.^20^, ^21^, ^25^

In this study, ARDS was the most common complication among immunocompromised patients. SARS‐CoV‐2 could cause mild to severe respiratory disease, and 17%−29% of these cases lead to ARDS.^27^ Therefore, it is important to identify patients at higher risk of ARDS by monitoring the oxygen saturation (SpO_2_) and respiratory rate during hospitalization.^28^

In the present study, diffuse (12.2%) and anterior (77.0%) lung involvement were the most common radiologic patterns in immunocompromised patients. Similar to previous studies, the most common CT scan findings in COVID‐19 patients in the present study were ground‐glass opacity and consolidation.^28^, ^29^

Two factors that remained significantly associated with immunosuppression in multivariate analysis were recovery time and Lopinavir/Ritonavir (Kaletra). According to recent studies, recovery time is longer in immunocompromised COVID‐19 patients compared with immunocompetent COVID‐19 patients.^31^ Immunosuppression prolongs the course of coronavirus disease and delays virus clearance.^30^, ^31^ Although there have been several therapeutic regimens in COVID‐19 patients, such as IFN‐alpha, lopinavir/ritonavir (Kaletra), chloroquine phosphate, and Ribavirin,^21^, ^32^ in immunocompromised patients such as renal transplant recipients, Lopinavir/Ritonavir (Kaletra) (protease inhibitors) was the most commonly prescribed medication.^34^ However, in a recent study, Lopinavir/Ritonavir (Kaletra) did not prove to improve the clinical recovery, mortality, and detectability of viral RNA in throat swabs of COVID‐19 patients.^35^ Meanwhile, there was some evidence that indicates the effectiveness of Lopinavir/Ritonavir (Kaletra) in decreasing the 28‐day fatality, ICU admissions, and hospital discharging time.^36^

There are limitations in the present study, such as the limited number of immunodeficient patients reducing the study power. The medical records of hospitalized patients were used in the present study which included some missing data for some patients, which prevented the inclusion of some factors, such as the prehospital duration of symptoms, medication history, and underlying diseases in our analysis.

## 5 CONCLUSION

In conclusion, recovery time was significantly longer in the immunocompromised compared to the immunocompetent group, which emphasizes the necessity of prolonged care in these high‐risk patients. Also, it is recommended to investigate the effect of novel therapeutic interventions to reduce the recovery time in addition to improving the prognosis of immunodeficient patients with COVID‐19. Further, epidemiological studies are recommended to evaluate the effect of underlying diseases on the clinical course of immunocompromised COVID‐19 patients and discover new therapeutic approaches.

## AUTHOR CONTRIBUTIONS

**Ladan Abasian, Fatemeh Jafari, SeyedAhmad SeyedAlinaghi, Niloofar Ayoobi Yazdi, Morteza Daraei, Nasrin Ahmadinejad, Fereshteh Ghiasvand, Hosein Khalili, Arash Seifi, Mohsen Maydani, Farzane Behnezhad, and Zahra Ahmadinejad**: Performed the research study, contributed to data accusation, investigation, and software. **Ladan Abasian and Zahra Ahmadinejad**: Designed the research study, prepared original draft and reviewed, and edited the final manuscript. **Zahra Ahmadinejad and Fatemeh Jafari**: Revised and prepared final manuscript. All authors collaborated in manuscript writing and analysis, with Zahra Ahmadinejad acting as an observer. All authors reviewed the manuscript.

## CONFLICT OF INTEREST STATEMENT

The authors declare no conflict of interest.

## ETHICS STATEMENT

The ethical necessities of the study were reviewed and approved by the Institutional Review Board (IRB) of the Tehran University of Medical Sciences with the file number (IR.TUMS.VCR.REC.1399.053). The ethical obligations were considered to respect the patient's rights and ensure data confidentiality.

## Data Availability

The data that support the findings of this study are available from the corresponding author upon reasonable request.
